# Unexpected Transcripts in Tn7 *orf19.2646 C. albicans* Mutant Lead to Low Fungal Burden Phenotype *In vivo*

**DOI:** 10.3389/fmicb.2017.00873

**Published:** 2017-05-16

**Authors:** Aude Pierrehumbert, Françoise Ischer, Alix T. Coste

**Affiliations:** Institute of Microbiology, University of Lausanne and University Hospital CenterLausanne, Switzerland

**Keywords:** *Candida albicans*, Tn7 mutant, unexpected transcript, virulence, fungal burden

## Abstract

The commensal fungus *Candida albicans* is the major cause of fungal systemic infection in immuno-compromised patients, with a mortality rate approaching 50% in the case of bloodstream infections. There is therefore a clear need to better understand fungal biology during infection to improve treatment. One of the particularities of *C. albicans* is its capacity to adapt to drastically diverse environments such as brain, bloodstream or gut. Adaptations to environmental change are mediated by transcription factors (TF) that modulate the expression of their target genes. Previous screening of a collection of Tn7 *C. albicans* TF mutants *in vivo* identified *orf19.2646* as playing a crucial role in the ability of the fungus to survive within its host. Indeed, the *orf19.2646* Tn7 interruption mutant strain displayed a reduced fungal burden compared to the wild-type strain. Surprisingly, an independent deletion mutant did not recapitulate the phenotype of the Tn7 interruption mutant. In the present study, we therefore investigated the difference between these two mutants and determined by performing a RACE analysis whether unexpected transcripts of the Tn7 mutant occurred. We found that two such transcripts upstream and downstream of the Tn7 insertion site were produced. The two transcripts were expressed in an *orf19.2646* deletion mutant which displayed a significantly reduced fungal burden level compared to the wild-type in *G. mellonella*. When the regions corresponding to these transcripts were deleted in the Tn7 mutants, the strains lacking both regions displayed a fungal burden similar to that of the wild-type strain. This study shows for the first time that mRNA transcription may occur downstream of a Tn7 sequence. In addition, these results demonstrated that the low fungal burden phenotype observed in the *orf19.2646* Tn7 mutant is due to the presence of these two transcripts together participating to an unidentified virulence mechanism to be further elucidated.

## Introduction

*Candida albicans* is one of the most successful fungal pathogens and is responsible for more than 50% of all *Candida spp*. infections (Wisplinghoff et al., 2014). *Candida* infections are opportunistic infections occurring in immunosuppressed patients or patients with risk factors such as invasive surgery (patients in Intensive Care Units), broad spectrum antibiotherapy, or the use of catheters. Even if such patients are treated with antifungal drugs either prophylactically or because of an established infection, once the infection reaches the bloodstream and becomes systemic, prognosis is poor, with a mortality rate of up to 50% (McNeil et al., 2001; Gudlaugsson et al., 2003; Lortholary et al., 2014; Puig-Asensio et al., 2014). Even though crucial virulence factors have already been identified, such as filamentation (Braun and Johnson, 2000; Klein and Tebbets, 2007; Fuchs et al., 2010), biofilm formation (Harriott and Noverr, 2011; Akers et al., 2015; Nobile and Johnson, 2015; Rajendran et al., 2016), and iron level adaptation (Chen et al., 2011; Chen and Noble, 2012; Noble, 2013), a further understanding of the fungal factors necessary to successfully infect the host is urgently needed. For this reason, we have previously assessed the role of *C. albicans* transcription factors (TFs) in the mouse bloodstream and *G. mellonella* infection models, using a collection of around 300 TF mutants (Vandeputte et al., 2011; Amorim-Vaz et al., 2015). This collection was achieved using a genomic library transposed with a Tn7 transposon flanked by a UAU cassette (Nobile and Mitchell, 2009). All plasmids thus obtained were sequenced at The Institute for Genomic Research, Rockville, MD (TIGR) consortium to determine the site of Tn7 insertion, and then used to generated *C. albicans* mutants (Nobile and Mitchell, 2009).

We initially focused our efforts on the Zn2Cys6 TF family (Vandeputte et al., 2011). Around 80 mutants were screened in a murine disseminated infection model. Groups of mice were infected with pools of 10 barcoded strains, consisting of 8 mutants, plus one isogenic wild-type strain and one avirulent isogenic *cmp1* mutant as controls. The relative proportion of mutants was measured by quantitative PCR (qPCR). This screening revealed that different strains displayed either hypo- or hyper-kidney fungal burden phenotypes as compared to the wild-type strain (Vandeputte et al., 2011). In such pools of strains, the competitive fitness of strainsplays a role in the overall virulence. Strains showing a significantly reduced or increased fungal burden were then tested again in single strain infections to eliminate this “pool effect” (Vandeputte et al., 2011). Finally, 3 mutants were found to display a significantly reduced fungal burden in the murine kidney as compared to the wild-type strain: *orf19.2646* (*ZCF13*), *orf19.3405* (*ZCF18*), and *orf19.3753* (*SEF1*). One of these mutants, *orf19.2646* was of particular interest since it exhibited no growth deficiency *in vitro* and was not previously described (Vandeputte et al., 2011). To validate the observed phenotype, a revertant strain of the *orf19.2646* Tn7 insertion mutant was also constructed. The re-introduction of a wild-type allele abolished the low fungal burden phenotype, thus confirming the role of *orf19.2646* in this phenotype (Vandeputte et al., 2011). The low fungal burden phenotype was confirmed in our subsequent study in *Galleria mellonella* single strain infections (Amorim-Vaz et al., 2015). One caveat when using the Tn7-UAU cassette in a given gene is that the deduced ORF is interrupted and not deleted. In addition, the *URA3* and *ARG4* auxotrophic markers of the cassette are ectopically expressed. Indeed, auxotrophic markers such as *URA3* have been shown to play a role in virulence (Brand et al., 2004). An independent mutant for *orf19.2646* was therefore produced by complete deletion of the gene using a *SAT1*-flipper recyclable cassette, leading to the removal of the selection marker from the genome (Amorim-Vaz et al., 2015). This mutant was assessed by kidney fungal burden in mice and fungal burden larvae in *G. mellonella*. Surprisingly, this null mutant showed no significant reduced tissue colonization as compared to a wild-type strain (Amorim-Vaz et al., 2015). Taken together, these data suggest that the reduced kidney fungal burden phenotype of the mutant was dependent on the gene inactivation approach taken, either by Tn7 insertion or deletion, suggesting that some interfering factors played an additional role. It has been previously shown that chimeric transcripts can be produced upon transposon insertion in filamentous fungal species, such as *Magnaporthe grisea* and *Mycosphaerella graminicola* (Lo et al., 2003). These transcripts were described as containing a part of the Tn7L or Tn7R. Indeed, stop codons are present in all frames of Tn7L or Tn7R, thus causing premature polyadenylation of the transcript located upstream of the mutated ORF. We therefore addressed whether chimeric *orf19.2646* transcripts could be produced in the Tn7-interrupted mutant and whether they could be responsible for the low fungal burden phenotype of the Tn7 insertion mutant.

## Materials and methods

### Strains, plasmids, and growth conditions

*Escherichia coli* DH5α was used as a host for plasmid propagation. To grow DH5α, Luria-Bertani (LB) broth medium was used and when necessary, supplemented with ampicillin (0.1 mg/ml). Cultures of *E. coli* were incubated at 37°C under constant agitation (220 rpm) for 16–20 h. For plate cultures, 0.7% BactoTM Agar (Brunschwig, Switzerland) was added.

All *C. albicans* strains used in this study are listed in Table 1. *C. albicans* were grown in complete Yeast Extract Peptone Dextrose (YEPD) medium (1% Bacto peptone, 0.5% yeast extract and 2% glucose) at 30°C under constant agitation (220 rpm). For growth on plates, 2% BactoTM Agar was added to the medium. Yeast cells were transformed by a lithium-acetate procedure with slight modifications as previously described (Sanglard et al., 1996). After addition of Salmon sperm DNA (0.137 mg/ml, Invitrogen) and heat shock steps, nourseothricin-resistant transformants were resuspended in 1 ml of YEPD medium and incubated for at least 4 h at room temperature. The cells were harvested in 100 μl of YEPD and plated on YEPD agar plates containing 200 μg/ml nourseothricin (Werner Bioagents, Germany).

**Table 1:** **List of strains**.

	**Strain**	**Name in the text**	**Genotype**	**Genetic background**	**References**
BWP17 background	ACY293	WT collection	BWP17 *ACT1*::pDS1551	BCY31	Vandeputte et al., 2011
	ACY294	Tn7 mutant	*orf19.2646*::Tn7-UAU, *ACT1*::pDS1551 *orf19.2646*::Tn7-*URA3*	BCY152	Vandeputte et al., 2011
	ACY292	WT revertant	*orf19.2646*::Tn7-UAU, *ACT1*::pAC249 *orf19.2646*::Tn7-*URA3*	BCY152	Vandeputte et al., 2011
	ACY413		*orf19.2646*::Tn7; Tn7 upstream; Tn7 downstream::(pAC321) FRT-*SAT1*-FRT/… *orf19.2646*::Tn7; Tn7 upstream; Tn7 downstream	BCY152	This study
	ACY417		*orf19.2646*::Tn7; Tn7 upstream; Tn7downstream::FRT/ *orf19.2646*::Tn7; Tn7 upstream; Tn7 downstream	ACY413	This study
	ACY420		*orf19.2646*::Tn7; Tn7 upstream::(pAC323) FRT-*SAT1*-FRT; Tn7downstream::FRT/ *orf19.2646*::Tn7; Tn7 upstream; Tn7 downstream	ACY417	This study
	ACY421		*orf19.2646*::Tn7; Tn7 upstream::FRT; Tn7downstream::FRT/ *orf19.2646*::Tn7; Tn7 upstream; Tn7 downstream	ACY420	This study
	ACY422	Up deletion	*orf19.2646*::Tn7; Tn7 upstream::FRT; Tn7downstream::FRT/ *orf19.2646*::Tn7; Tn7 upstream::(pAC323) FRT-*SAT1*-FRT/…; Tn7downstream	ACY421	This study
	ACY423		*orf19.2646*::Tn7; Tn7 upstream::FRT; Tn7downstream::FRT/ *orf19.2646*::Tn7; Tn7 upstream::FRT; Tn7downstream	ACY423	This study
	ACY428	Up + Do deletion	*orf19.2646*::Tn7; Tn7 upstream::FRT; Tn7downstream::FRT/ *orf19.2646*::Tn7; Tn7 upstream::FRT; Tn7downstream::(pAC321) FRT-*SAT1*-FRT/…	ACY423	This study
	ACY430	Do deletion	*orf19.2646*::Tn7; Tn7 upstream; Tn7downstream::FRT/ *orf19.2646*::Tn7; Tn7 upstream; Tn7downstream::(pAC321) FRT-*SAT1*-FRT/…	ACY417	This study
SC5314 background	SC5314				
	ACY297		*orf19.2646*/*orf19.2646* Δ	SC5314	Amorim-Vaz et al., 2015
	ACY315	Deletion mutant	*orf19.2646* Δ/Δ	SC5314	Amorim-Vaz et al., 2015
	ACY317	WT revertant	*orf19.2646* Δ/Δ, *ACT1*::(pAC253-3) FRT-*SAT1*-FRT	ACY315	Amorim-Vaz et al., 2015
	ACY339	S-revertant	*orf19.2646* Δ/*orf19.2646* Δ::(pAC339) FRT-*SAT1*-FRT	ACY315	This study
	ACY352		*orf19.2646* Δ/*orf19.2646* Δ::pAC339 FRT	ACY339	This study
	ACY362	S + WT revertant	*orf19.2646* Δ::(pAC253) FRT-*SAT1*-FRT/*orf19.2646* Δ::(pAC339) FRT	ACY352	This study
	ACY401		*orf19.2646*/*orf19.2646*::pAP3	SC5314	This study
	ACY402	Up-revertant	*orf19.2646* Δ/*orf19.2646* Δ::pAP3	ACY315	This study
	ACY403		*orf19.2646*/*orf19.2646*::pAP4	SC5314	This study
	ACY404	Do-revertant	*orf19.2646* Δ/*orf19.2646*::pAP4	ACY297	This study
	ACY405	Up + Do-revertant	*orf19.2646*::pAP3/*orf19.2646*::pAP4	ACY401	This study

All plasmids and primers used in this study are listed in Tables 2, 3, respectively. For pAC339, the first 885 bp of *orf19.2646* was amplified and 1,000 bp upstream of the ATG using primers 2646-promF-KPN and the 2646-insert2-xho (Table 3). This PCR product was cloned in pAC245 (Table 2) between KpnI and XhoI sites replacing the 1,000 bp upstream sequence by the truncated version of *orf19.2646* flanked by 1,000 bp upstream sequence. This construct was then transformed after a digestion by KpnI-SacI in ACY315 (*orf19.2646*Δ/Δ) leading to strain ACY339. For the construction of pAP3, a PCR was performed using primers 2646-promF-KPN and 2646-postRACE5′Reverse (Table 3) on the genomic DNA of ACY294 to amplify the region corresponding to the upstream transcript and cloned in pAC245 at XhoI-KpnI sites. This construct was then transformed after a digestion by KpnI-SacI in ACY315 (*orf19.2646*Δ/Δ) and SC5315 yielding to ACY402 and ACY401, respectively, in which the deleted allele of *orf19.2646* was replaced by the upstream transcript cassette by double cross-over. For the construction of pAP4, a PCR was performed using primers 2646-postRACE3′Forward and 2646-postRACE3′Reverse (Table 3) on the genomic DNA of ACY294 to amplify the region corresponding to the downstream transcript and cloned in pAC245 at SacI-SacII sites. This construct was then transformed after a digestion by KpnI-SacI in ACY297 (*orf19.2646*Δ/*ORF19.2646*) and SC5315 yielding ACY404 and ACY403, respectively. For pAC323 used to delete the region encoding the Tn7 upstream transcript, a PCR was performed on the 3′-end of the upstream transcript using primers 2646Del5′_R-bis-SacI and 2646Del5′_F-bis-SacII (Table 3) on BCY152 genomic DNA and cloned in pAC245 at SacI-SacII sites. For pAC321 used to delete the region encoding the Tn7 downstream transcript, a PCR was performed on the 5′-end of the downstream transcript using primers Deletion-3′-F-KpnI and Deletion-3′-R-Xho (Table 3) on BCY152 genomic DNA of and cloned in pAC245 at KpnI and XhoI sites. All these plasmids were digested by KpnI and SacI before transformation in yeast strains. To delete the second allele of the Tn7 upstream or downstream regions, the *SAT1* cassette was regenerated by the use of *FLP* recombinase, which is controlled through a maltose-inducible promoter (Reuss et al., 2004).

**Table 2:** **List of plasmids**.

**Plasmid**	**Characteristics**	**References**
pAC245	*orf19.2646* deletion cassette	Amorim-Vaz et al., 2015
pAC253	*orf19.2646* WT revertant cassette	Amorim-Vaz et al., 2015
pAC339	*orf19.2646* 885 bp revertant cassette	This study
pAP3	pAC245/Tn7 upstream CDS	This study
pAP4	pAC245/Tn7 downstream CDS	This study
pAC321	pAC245/5′ part of the downstream transcript	This study
pAC323	pAC245/3′ part of the upstream transcript	This study

**Table 3:** **List of primers**.

**Name**	**Sequence (5′−3′)**
2646-start-Hind	GCGCAAAAGCTTATGGATAAGACAAATAGTCCAGGC
2646-insert1-xho	CGCGAACTCGAGAATTGATAATGGTTGGTGCTG
2646-insert2-xho	CGCGAACTCGAGAATACTCATGCCAGATATTGATG
2646-insert3-xho	CGCGAACTCGAGAATTGGAGCTGTTTGATCAGATAAC
2646-stop-sph	GCGCAAGCATGCTTATAGAAGATCATTGAAATCACC
2646-Sybr-RevB	GCACTCATGGCATCGGTGGCT
2646-promF-KPN	GCGCAAGGTACCGATATAACAATTATTTGTACACC
2646-+500	CCGCCACCACCACCG
2646-up-F	TCGGTCTAGTGCATCTCCTCA
2646-up-R	CTGCTGGAAATGGTGGTTGTG
2646-up-probe	ACCAACCACCGCCAATACAT
2646-do-F	CCACTCGTTACACCACATTTG
2646-do-R	TTCACCCGTGGGATCTTC
2646-do-probe	CCGATGCCATGAGTGCATTG
Act1-F	ATAACGGTTCTGGTATGT
Act1-R	CCTTGATGTCTTGGTCTA
Act1-probe	CGGTGACGACGCTCCAAG
2646-P1-RACE	AACCACAACCACCATTTCCAGCAGCAGCA
2646-P1b-RACE	AGGCACAAATCTCCATTCCAACGCCACCA
2646-P2b-RACE	TTTAGTAGCCACCGATGCCATGAGTGCA
2646-P3-RACE	AACGTGCGGGTATTCAACATCACACCCA
2646-P3b-RACE	AAGATGCACTGCTGTTGCCGTTACCGTTG
2646-P4-RACE	TGGTGGCGTTGGAATGGAGATTTGTGCCT
2646-P4b-RACE	TGCTGCTGCTGGAAATGGTGGTTGTGGTT
GeneRacer oligo dT primer	GCTGTCAACGATACGCTACGTAACGGCATGACAGTG(T)_24_
GeneRacer 5′ primer	CGACTGGAGCACGAGGACACTGA
GeneRacer 5′ nested primer	GGACACTGACATGGACTGAAGGAGTA
GeneRacer 3′ primer	GCTGTCAACGATACGCTACGTAACG
GeneRacer 3′ nested primer	CGCTACGTAACGGCATGACAGTG
2646-postRACE5′reverse	CGCAAACTCGAGCCGTTTATACCATCCAAATC
2646-postRACE3′forward	CGCAAACCGCGGCACGCATCTTCCCGACAACG
2646-postRACE3′reverse	CGCAAAGAGCTCGCAGGTTTACAAACCACATC
orf19.2646-up5	TCAATCAAGCCTCCTGTACCACCAC
orf19.2646-down3	CTCATTATTAGGAGTTGCTAACCA
Primer N	ACTTTATTGTCATAGTTTAGATCTATTTTG
Primer S	TATTAGGAATTTTTGAGGTAAAGGTGGGGA
UAU-amplif-R	CTGTGCTACTGGTGAGG
UAU-seq-R	GTCTTAGTGTTGACTGTC
2646-seq-5	ATGGAGTGTTGTCACC
2646Del5′_R-bis-SacI	CGCGAGCTCTATAGCCTCCATTAGATC
2646del5′-F-bis-SacII	CGCCCGCGGCTAATTTCAATCCTAGCAC
Deletion-3′-F-KpnI	AAACGCGGTACCAGAATTCTAATCCAACGG
Deletion-3′-R- Xho	AAACGCCTCGAGGATCTGATTGATATTAAACTC

### RNA extraction RT-qPCR analysis

*C. albicans* strains were grown overnight in 3 ml YEPD at 30°C under agitation. Fifty microliters of cultures were diluted in 5 ml YEPD and incubated at 30°C until reaching 1.5 × 10^7^ cells/ml. The cultures were then centrifuged 5 min at 13,000 rpm. The pellet was resuspended in 350 μl RNA buffer (0.1 M Tris HCl pH 7.5, 0.1 M LiCl, 10 mM EDTA, 0.5% SDS) and transferred into tubes with screwed caps containing 200 μl diethylpyrocarbonate (DEPC)-treated beads and 300 μl phenol/chloroform/isoamyl alcohol (25:24:1). The tubes were shaken using the bead beater FastPrep-24 (MP biomedicals, Santa Ana, CA) with the following settings: speed 5 m/s and time 5 s. The tubes were next centrifuged for 1 min at full speed. The supernatant was transferred into tubes containing 250 μl phenol/chloroform/isoamyl alcool (25:24:1) and tubes were vortexed for 10 s and centrifuged for 1 min at full speed. The supernatant was transferred into tubes containing 600 μl ethanol (EtOH) 100% and kept at −20°C for 30 min (or alternatives: 10 min in dry ice). The tubes were centrifuged for 2 min at 4°C and full speed. The RNA pellet was washed with 600 μl EtOH 70%. The pellets were air-dried and resuspended in 50 μl DEPC-water. An RNase inhibitor (RNasin®, Promega, Switzerland) was added to the samples and they were stored at −80°C.

After RNA extraction and DNase treatment, the integrity of the RNA was verified using a BioAnalyzer (Agilent Technologies, Waldbronn, Germany).

RNA was first reverse transcribed into cDNA using the Transcriptor High Fidelity cDNA synthesis kit (Roche Applied Science, Penzberg, Germany). Briefly, 10 ng total RNA was added to Oligo dT (60 μM) in a total volume of 11.4 μl. The mix was incubated for 10 min at 65°C and the tubes were stored back on ice. Reaction buffer (1X), deoxynucleotide mix (2 μM), 1,4-Dithiothreitol (DTT; 10 μM), protector RNase inhibitor (20U), and transcriptor high fidelity reverse (1U) were added to the mix, incubated for 30 min at 50°C and inactivated for 5 min at 85°C. The qPCR was performed using 100-fold diluted cDNA, Taq supermix with ROX (1X) (BioRad, Hercules, CA), primers, and probe at 100 nM final concentration each. The two probes used, e.g., upstream (up-) and downstream (do-) (Table 3), were Taqman probes (FAM-TAMRA). StepOnePlus software was applied to analyse data (Applied Bioscience, Foster city, CA). Relative mRNA levels were calculated as previously described (Sierro et al., 2001) using the *ACT1* housekeeping gene (Table 3).

### RNA ligase-mediated-rapid amplification of cDNA ends (RLM-RACE)

The RLM-RACE was performed according to manufacturer's protocol (GeneRacer™ Kit, Thermofisher, Switzerland). Briefly, total RNA (5 μg) from ACY293 and ACY294 were used to perform the RACE analysis. HeLa total RNA (1 μg) was used as internal control of the kit. The three samples were first treated with calf intestinal phosphatase (CIP) in order to dephosphorylate RNAs that are either truncated mRNA with a polyA-tail but not 5′ capped or other non-mRNA. The dephosphorylated RNA was then precipitated before being treated with tobacco acid pyrophosphatase (TAP) to remove the 5′ cap structure of the mRNA, thereby exposing the 5′ phosphate. The RNA was next ligated with GeneRacer RNA Oligo to the 5′ phosphate using T4 RNA ligase. The RNA was reverse transcribed using a GeneRacer Oligo dT Primer. This resulted in the creation of a RACE-ready first-strand cDNA. GeneRacer 5′ primers and reverse gene specific primers (Reverse GSP) were used to amplify the 5′ends, while GeneRacer 3′ primers and forward gene specific primers (Forward GSP) were used to amplify the 3′-ends. The PCR was performed using the hot start Platinum Taq DNA Polymerase High Fidelity. The annealing temperature was 60°C and the elongation time varied depending on the expected product's length. If needed, GeneRacer nested primers that are located closer to the gene of interest were provided. Other GSP closer to the 5′- or 3′-ends were used to obtain a PCR product in combination with the GeneRacer primers. The PCR products were purified using the Nucleospin Gel and PCR Clean-up (Macherey-Nagel, Switzerland) and were ready for cloning.

PCR products were cloned into pCR™4-TOPO® TA vectors (Thermo Fisher Scientific, Switzerland) following instruction of the manufacturer. For the upstream transcript, GR5nested-P4b, GR3-P1, and GR3-P1b yielded Topo6-28, Topo2-6, and Topo3-11 plasmids respectively. For the downstream transcript, GR5nested-P3b, GR3-P2b yielded Topo8-36, -39, -40, and Topo4-16, -17, -19, respectively.

### Sequencing

Sequencing of plasmids was performed using an ABI Prism 3130 XL automated DNA sequencer (Perkin-Elmer/Applied Biosystems, Foster City, CA) with a BigDye terminator cycle sequencing kit (version 1.1, Applied Biosystems) according to the manufacturer's protocol using primers from the GeneRacer™ Kit (M13F, M13R, T3, and T7) diluted 20-fold (5 μM).

### Mice infection and ethic statement

All animal experiments were performed at the University Hospital Center of Lausanne with approval through the Institutional Animal Use Committee, Affaires Vétérinaires du Canton de Vaud, Switzerland (authorization n° 1734.3), according to decree 18 of the federal law on animal protection. For all mice experiments, female BALB/c mice (6 weeks old; Charles River France) were housed in ventilated cages with free access to food and water. The strains of *C. albicans* were grown in individual tubes for 16 h under agitation at 30°C in YEPD medium. Each strain was subsequently diluted 100-fold in YEPD medium and grown overnight under agitation at 30°C. Overnight cultures were washed twice with PBS (137 mM NaCl, 2.7 mM KCl, 10 mM Na2HPO4, 1.8 mM KH2PO4) and resuspended in 5 ml PBS. The concentration of each culture was measured through optical density, and each strain was diluted to 8 × 10^5^ cells/ml. The mice were injected through the lateral tail vein with 250 μl of cell suspension. At 3 days post-infection (dpi), the kidneys were recovered, and the Colony-forming units (CFU) were determined as previously described (Vandeputte et al., 2011). The strains were tested in groups of 5 mice and repeated twice. Experiment results were pooled and thus expressed as percent of CFU obtained with the isogenic wild-type strain used in each experiment. The limit of detection was 50 CFU per 2 kidneys.

### Galleria mellonella infection

*Galleria mellonella* fungal burden were assessed as described previously (Amorim-Vaz et al., 2015). Briefly, the different strains were grown overnight in 5 ml YEPD. The cultures were next washed in PBS and re-suspended in PBS complemented with 200 μg/ml ampicillin, at 5 × 10^6^ cells/ml. Each larva (350–400 mg) was injected through the last left proleg with 40 μl of inoculums (2 × 10^5^ cells). After 24 h in the dark at 30°C, each larva was introduced into screw-cap tubes containing a metal bead (stainless steel, 7 mm, [VWR International]). The tube was closed and firmly agitated performing three 10 s cycles at 6.5 m/s with a MP FastPrep®-24 equipment (MP Biomedicals). The homogenate was resuspended in 5 ml PBS in a 14 ml Falcon tube, serially 10-fold diluted in PBS. One hundred microliters were plated on YEPD-chloramphenicol (50 μg/ml) plates and incubated at 30°C for 24–48 h. CFU were then enumerated on three plates and means were calculated. Fungal loads were expressed as CFU per larvae. The limit of detection was 50 CFU per larvae. For feasibility of the experiments, all the mutants of one analysis could not be performed at the same time due to the high number of individual to manage. Several experiments were performed with different mutants always including the isogenic wild-type strains as a positive control. Some level of variability was observed from one experiment to another even with the same mutants, therefore data of fungal burden are always expressed as a percentage of the isogenic wild-type strain present in each individual experiment.

## Results

### Analysis of the *Orf19.2646* Tn7 interrupted mutant of the collection

**Figure 1:**
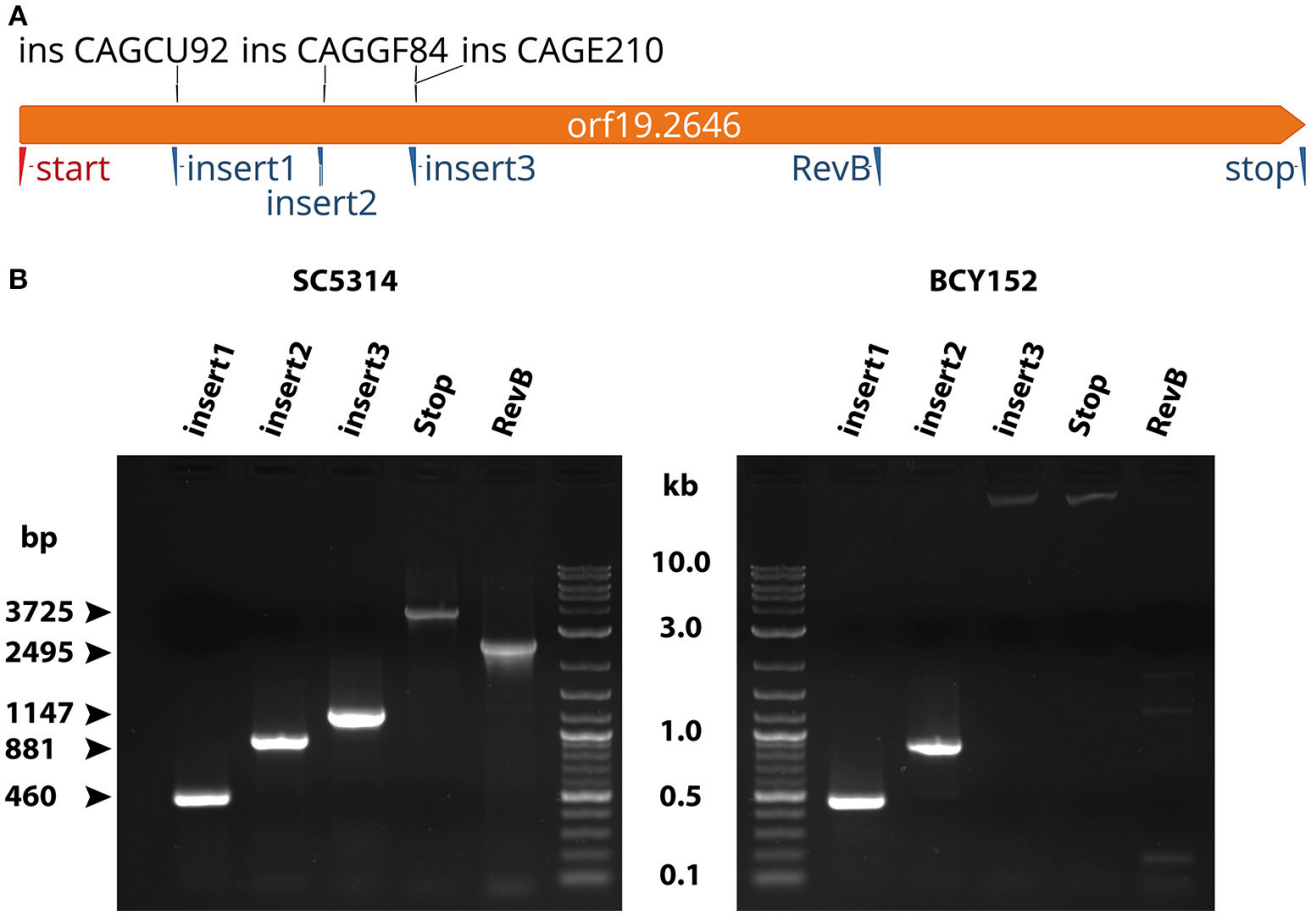
**Analysis of the Tn7-UAU cassette location in *orf19.2646*. (A)** Map of *orf19.2646* gene with the three different possible insertions (ins CAGCU92, ins CAGGF84, and ins CAGE210) of the Tn7-UAU cassette as sequenced by TIGR. Triangles represent position of the forward (red) and reverse (blue) primers used to detect the insertion of the Tn7-UAU cassette. **(B)** PCR amplification on SC5314 DNA (left panel) and orf192646 Tn7 mutant (BCY152) DNA (right panel). Each PCR was performed using the forward primer “start” and reverse primers as indicated.

In the TIGR database (Chamilos et al., 2009), 3 different mutants for *orf19.2646* were identified. Three possible insertions were described: ins CAGCU92 (position +460), ins CAGGF84 (position +885), and ins CAGE210 (position +1151) (Figure 1A). To determine which mutant was corresponding to strain BCY152 of the TF mutant collection (Vandeputte et al., 2011; Amorim-Vaz et al., 2015), a discriminative PCR was performed using a forward primer (orf19.2646-start-HindIII) with different reversed primers (2646-insert1, 2646-insert2, 2646-insert3, 2646-Sybr-RevB, and 2646-stop-Sph) (Figure 1A). As a positive control DNA from strain SC5314 was used. PCR products obtained using the reverse primers insert1 and insert2 yielded products of 460- and 881-bp for both DNA, respectively (Figure 1B). In contrast, PCR with insert3, RevB, and Stop primers yielded products of 1147-, 2495-, and 3725-bp for SC5314 DNA only (Figure 1B), respectively. For BCY152 amplification products were observed above 10 kb with reverse primers insert3 and RevB but no signals were observed in the presence of Stop primer (Figure 1B). These results indicate that the Tn7 cassette present in the BCY152 *orf19.2646* mutant was positioned between the insert2 and insert3 primers, thus corresponding to the CAGGF84 TIGR clones in which the Tn7 cassette was predicted to be inserted at position +885 relative to the ATG. Thus, the first 885-bp of *orf19.2646* are still present in this mutant and could lead to the production of a chimeric transcript. This truncated transcript might be responsible for the low fungal burden phenotype observed in previous studies (Vandeputte et al., 2011; Amorim-Vaz et al., 2015).

### Analysis of the *orf19.2646* deletion mutant carrying a truncated transcript corresponding to the first 295aa

**Figure 2:**
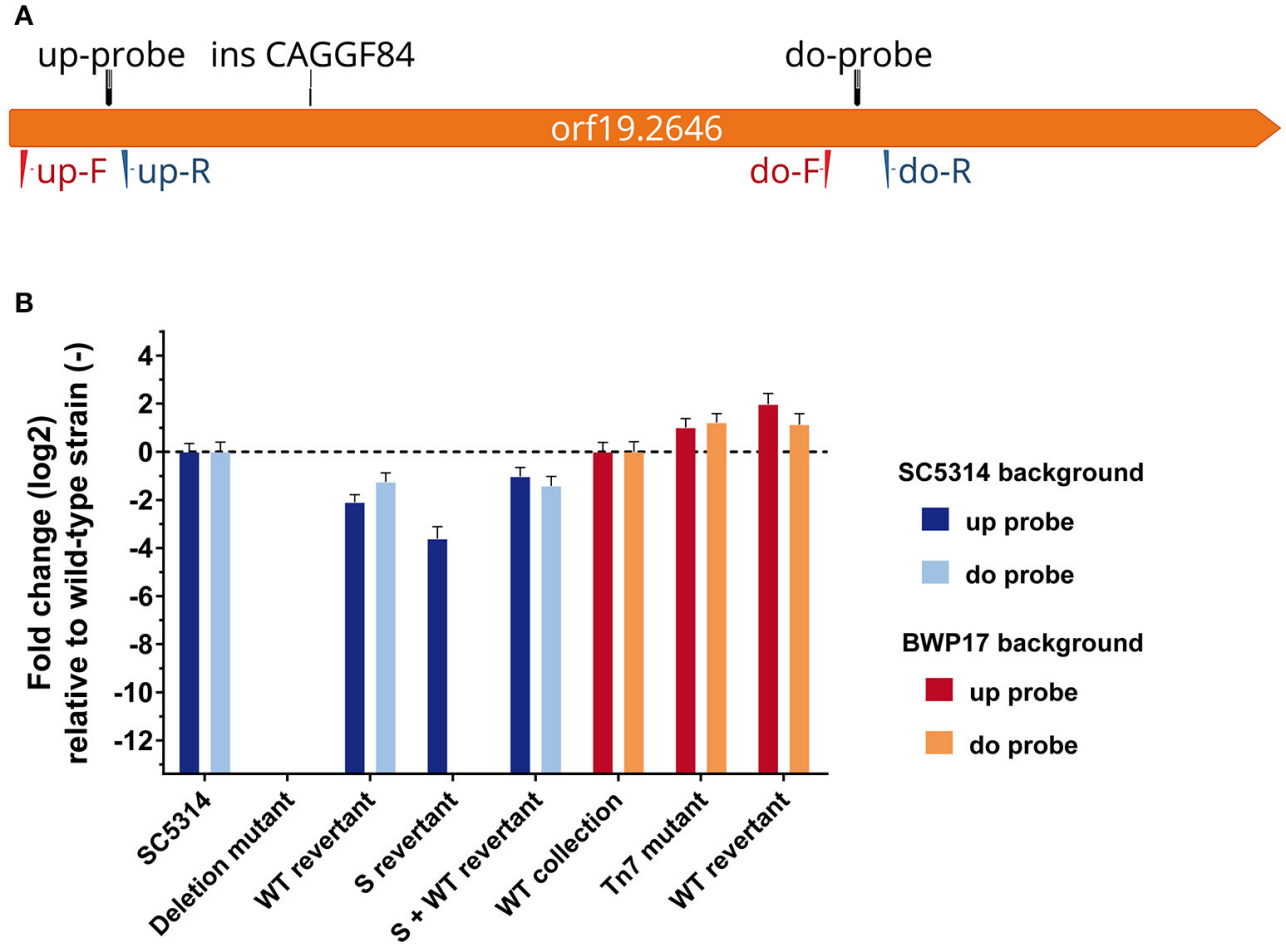
**mRNA level of Tn7 putative upstream and downstream transcript. (A)** Map of *orf19.2646* with the position of the upstream (up-) and downstream (do-) primers and up- and do-TaqMan™ probes. **(B)** RT-qPCR relative quantification of upstream and downstream transcripts. Results are expressed relative to the quantity measured for the isogenic wild-type strains. Each bar-plot corresponds to the mean of two biological samples measured in triplicate.

To test the above-mentioned hypothesis, we amplified the first 885-bp (corresponding to 295 aa of *orf19.2646* in addition to 1,000-bp upstream of *orf19.2646* ATG (using primers 2646-promF-KPN and 2646-insert2-xho) and cloned it into a cassette to allow recombination with *orf19.2646* in the deletion mutant. We called this truncated version of *orf19.2646* the “S” (short) allele. The S construct was integrated at the *orf19.2646* locus by homologous recombination in the deletion mutant. This strain was designated as “S-revertant” (Figure 2). In addition, a wild-type (WT) copy of *orf19.2646* was introduced by transformation of the pAC253 revertant cassette (Amorim-Vaz et al., 2015). This last strain was designated as “S + WT–revertant” (Figure 2).

As a first analysis, the expression of the construct was verified by RT-qPCR using primers and probes hybridizing upstream (up) and downstream (do) the insertion of the Tn7 cassette (up-F, up-R and up-probe, and do-F, do-R and do-probe, respectively) (Figure 2A). We tested the transcription of DNA upstream and downstream the first 885-bp in SC5314, the deletion mutant, the WT-revertant, and finally in the two new strains obtained in this study (S− and S + WT-revertants). We could clearly observe that the upstream probe gave a signal for all strains except the deletion mutant (Figure 2B), indicating that a transcript is produced in the S-revertant. In contrast and as expected, the Tn7 downstream probe gave a signal only for strains carrying a *orf19.2646* wild-type copy (SC5314, WT−, and S + WT-revertants; Figure 2B). We could also observe that the WT-revertant displayed a decreased *orf19.2646* expression as compared to SC5314. This can be explained by a gene dosage effect, since only one allele was re-introduced into the revertant strain.

**Figure 3:**
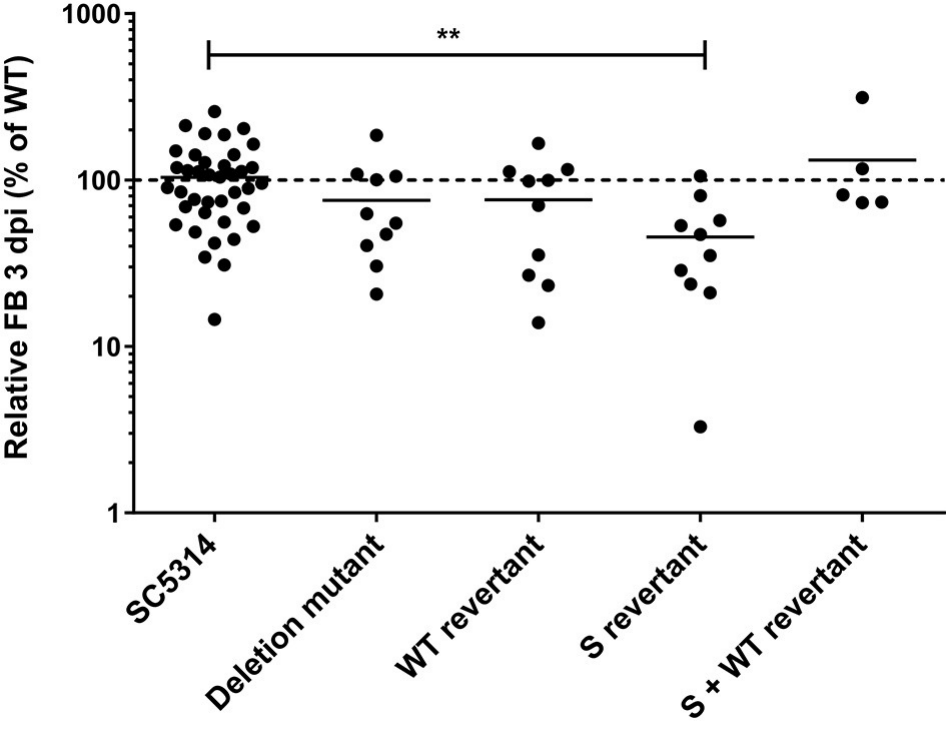
**Role of the 885 bp-truncated *orf19.2646* on mouse kidney fungal burden (FB)**. For each individual mouse, the FB in the kidneys is expressed as a percentage relative to the mean of the wild-type group in the same experiment. All experiments are pooled in this graph. The mean value of each group is indicated with a black bar. The dotted line indicates the level of the mean of the wild-type strain set at 100%. Each mutant was tested twice using five mice each time (except for S+WT revertant and SC5314 wild-type strains). Statistical analyses were performed using a Mann–Whitney test to assess CFU differences relative to SC5314 (GraphPad Prism 7.02). The stars indicate the level of statistical significance: ^**^p < 0.01. S stands for *orf19.2646* “short” version of 885 bp.

Next, the fungal burden phenotypes of all the five above described strains were tested in mice (Figure 3). The S-revertant, in contrast to SC5314, the deletion mutant and the WT-revertant, displayed lower fungal burden (Figure 3). This phenotype was reverted in this strain by an additional wild-type copy of *orf19.2646* (Figure 3, S+WT-revertant). This result is consistent with the hypothesis that an unexpected transcript corresponding to a truncated protein due to the Tn7 insertion might be responsible for the observed low fungal burden phenotype in the *orf19.2646* mutant of the collection.

### mRNA expression levels in the different strains

To further verify this hypothesis, the production of a transcript upstream of the Tn7 in the *orf19.2646* Tn7-UAU mutant was analyzed by RT-qPCR (Figure 2). The revertant strain and the isogenic wild-type strain were analyzed as controls (Figure 2B, BWP17 background). As expected in the wild-type and revertants strains, amplification at both the upstream and downstream Tn7 flanking regions could be observed. Amplification occurred at the upstream Tn7 flanking regions in the Tn7 mutant strain. Unexpectedly, we also observe an amplification product at the downstream region of the Tn7 mutant (Figure 2B). In the Tn7 mutant, the amount detected of both transcripts appeared to be higher than in the isogenic wild-type strain. This strongly suggests the presence of unexpected transcripts in the *orf19.2646* Tn7- mutant not only upstream the Tn7-UAU cassette insertion and corresponding to a truncated protein but also downstream of this cassette.

### Identification of Tn7 up-, and downstream transcript sequences

To further investigate the sequences of these two transcripts and to analyse whether they correspond to mRNA with all the features enabling the production of a protein, the Rapid amplification of cDNA ends (RACE) technique was used. This technique provides the sequence of an RNA transcript from a small known sequence within the transcript to the 5′- (5′ RACE-PCR) or 3′-end (3′ RACE-PCR) of the RNA. Performing both types of analysis provides the entire sequence of the putative Tn7 upstream and downstream mRNA.

**Figure 4:**
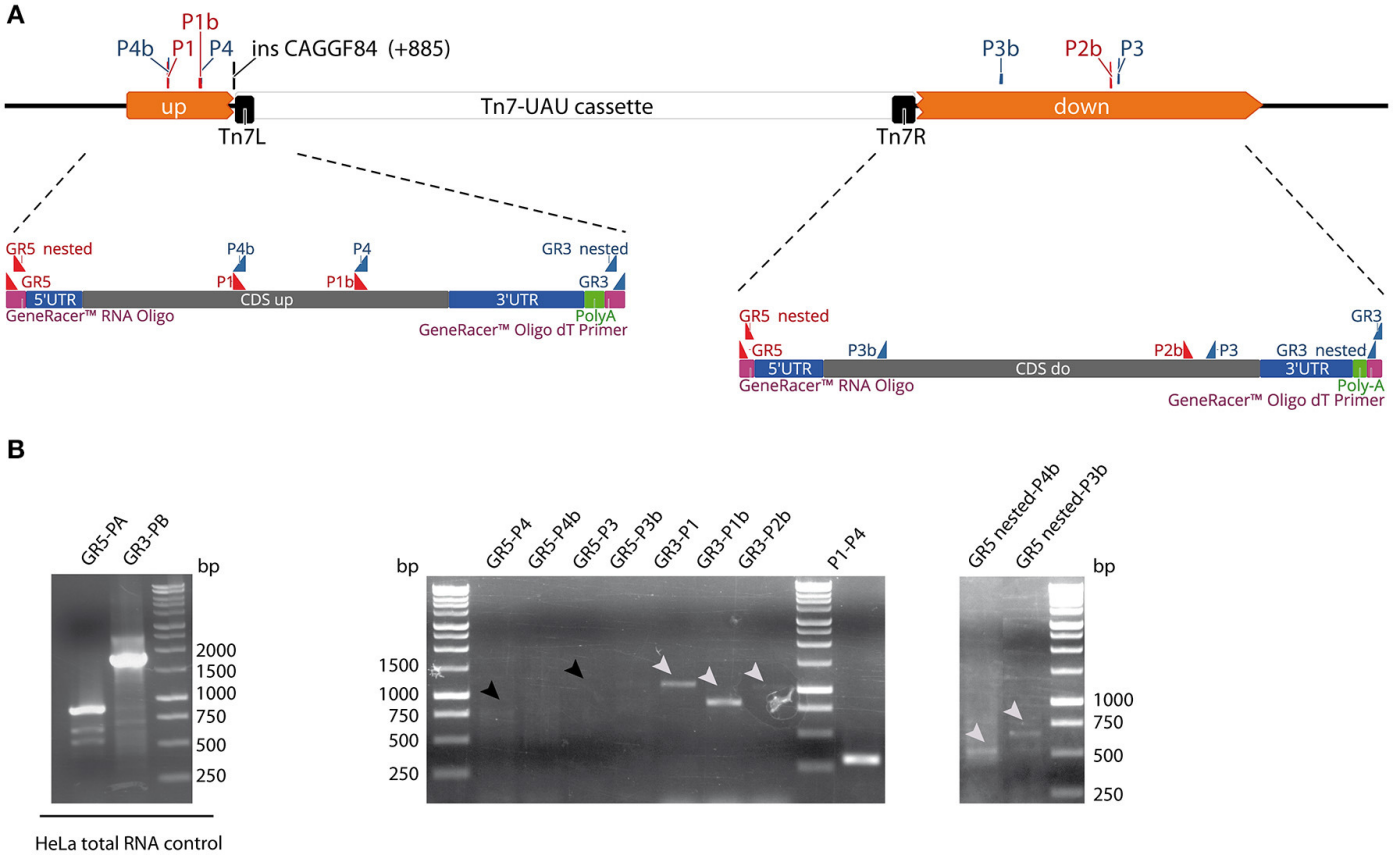
**RACE analysis. (A)** Map of Tn7 mutated *orf19.2646*. *Orf19.2646* (gray) is interrupted by the Tn7-UAU cassette (white bar). The putative transcripts drawn between the dotted lines have been ligated to a 5′ GeneRacer™ Oligo and 3′ GeneRacer™ Oligo dT primers (purple). The forward primers (P1, P1b, and P2b, red triangles) are designed to amplify a products with the GeneRacer 3′ primer (GR 3′), while the reverse primers (P3, P3b, P4, and P4b, blue triangles) are designed to amplify products with the GeneRacer 5′ primer (GR 5′). **(B)** Left panel: Control PCR with HeLa total RNA. Middle panel: RACE PCRs performed with the GR5′, the GR3′ primers and with the P1-P4 primers on SC5314 RNA as control of reverse transcription. Right panel: Nested PCR performed on the GR5-P4 and GR5-P3 PCR products (black arrows). Topo cloning was performed on the products indicated by a white arrow.

As shown in Figure 4A, the GSP forward primers (P1, P1b, and P2b) were used with the GeneRacer 3′ (GR3) primer for the 3′end cloning of the transcripts, while the GSP reverse primers (P3, P3b, P4, and P4b) were used with the GeneRacer 5′ (GR5) primer for the 5′end cloning of the transcripts. The strains used to perform the RACE analysis were the wild-type strain of the collection (ACY293) as a positive control and the Tn7 mutant (ACY294).

First, and to validate the procedure, the GeneRacer was carried out on RNA from HeLa cells with GSP allowing the amplification of the β-actin transcript. The expected sizes for both combinations of primers were obtained at 0.9 kb for the 5′-end and 1.8 kb for the 3′-end (Figure 4B, left panel), thus indicating that the RACE procedure was performed correctly. Second, to verify whether the whole mRNA was reverse transcribed correctly, a PCR was performed using the GSP primers P1 and P4. As expected these two primers were able to obtain a PCR product of 294 bp on the Tn7 upstream region (Figure 4B, middle panel), thus indicating that the reverse-transcription step was also correctly performed on the Tn7 mutant *C. albicans* RNA.

GR-GSP PCR was then performed to amplify the end of the putative Tn7 upstream and downstream transcript (Figure 4A). With regards to the amplification of the 3′-end of the mRNA, an amplification for both the upstream (GR3-P1 and GR3-P1b), and the downstream (GR3-P2b) transcripts was obtained. In the case of the upstream transcript, products of 1,000 bp for P1-GR3, and 800 bp for P1b-GR3 were observed (Figure 4B, middle panel). For the downstream transcript, a product of 1,000 bp was observed for P2b-GR3 (Figure 4B, middle panel). Regarding the 5′-end of this transcript, no amplification products were obtained for the upstream and downstream transcripts. We thus performed nested PCR on the product of P4-GR5 and P3-GR5 PCR products using internal primers P4b and P3b, respectively, with the GR5-nested primer provided in the RACE kit. Nested PCR yielded a product of 500 bp for the upstream transcript (P4b-GR5nested, Figure 4B, right panel) and of 700 bp for the downstream transcript (P3b-GR5nested, Figure 4B, right panel). Thus, both 5′- and 3′-ends of both transcripts were amplified. To determine the sequences of the transcript ends, the five PCR products were TOPO cloned and sequenced.

**Figure 5:**
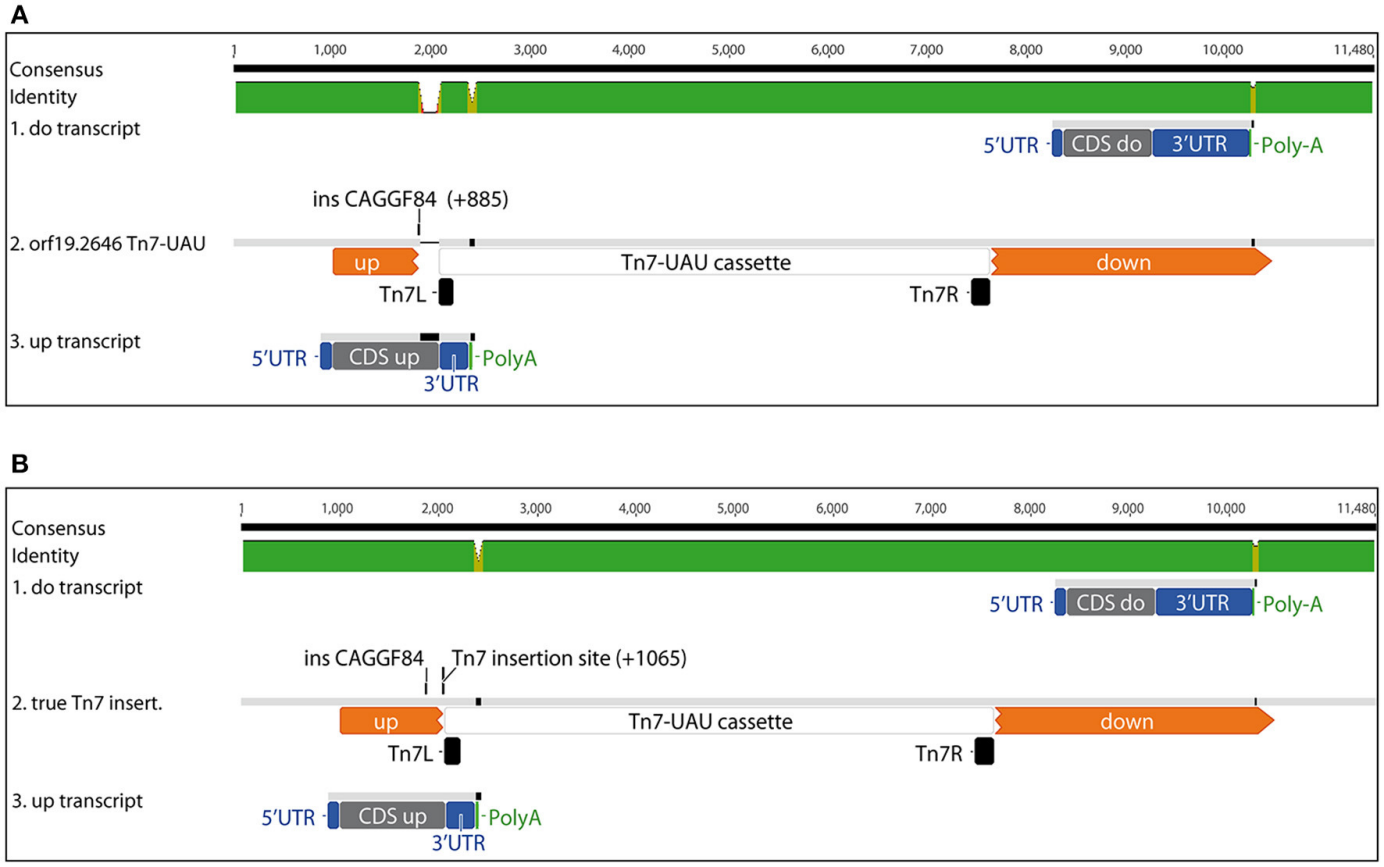
**Mapping of the upstream (up) and downstream (do) Tn7 transcripts. (A)** Mapping of the reconstituted upstream and downstream transcript sequences on the CAGGF84 TIGR sequence, **(B)** Newly deduced sequence for the *orf19.2646* Tn7-UAU insertion locus and alignment with the identified upstream and downstream transcripts.

The upstream transcripthas all the features of an mRNA (for complete sequences, see File S1): a 5′-untranslated region (UTR), a coding sequence (CDS), a 3′-UTR and a poly-A tail. Note that the cap could not be detected since it was removed during the RACE procedure. Then, the sequences were aligned together and with the *orf19.2646* Tn7 interrupted sequence as deduced from the TIGR description (Figure 5A). The two sequences did not perfectly align with the predicted *orf19.2646* Tn7 interrupted sequence. A gap of 180 bp was observed downstream of the TIGR described Tn7 insertion. It appeared that the Tn7 cassette was 180 bp downstream at position +1065 bp from the start of *orf19.2646*. After redesign of the map at the *orf19.2646* Tn7 interrupted locus, the upstream transcript could be aligned with this new sequence (Figure 5B). The start of the CDS is identical to *orf19.2646*. The stop codon is located in the Tn7R sequence. The 5′-UTR is part of the *orf19.2646* promoter and the 3′-UTR is situated at start of the UAU cassette.

The downstream transcript possessed also all features of an mRNA with 5′- and 3′-UTR, CDS, and PolyA sequences (see File S1). Then this downstream transcript was aligned with the *orf19.2646* Tn7 interrupted sequence (Figure 5). It aligned within the *orf19.2646* CDS, the Tn7L sequence being probably part of the promoter of this transcript (Figure 5).

### Reconstruction of the upstream and downstream transcript mutants

**Figure 6:**
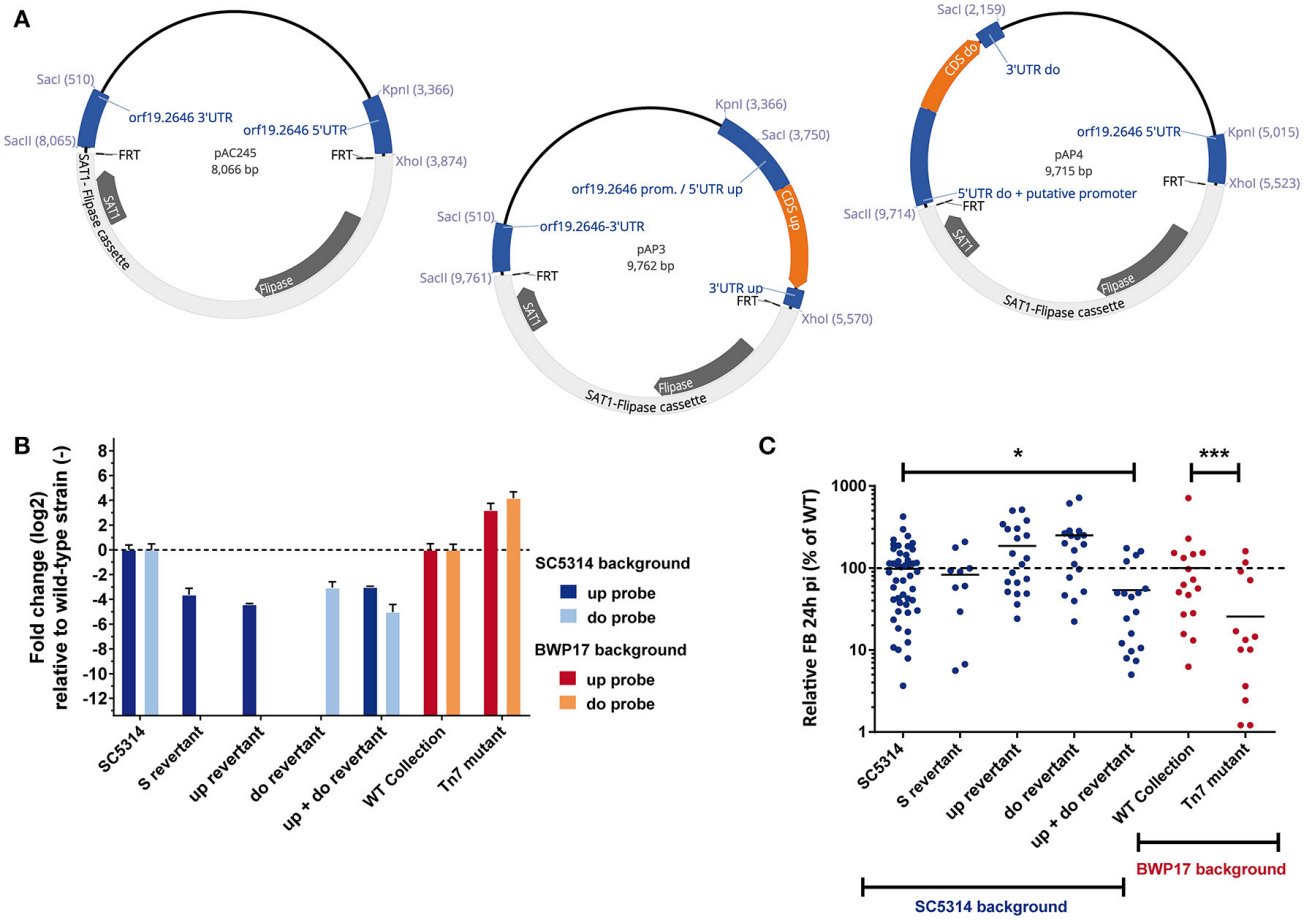
**Identification of the Tn7 up-, and downstream transcript sequences and role in the fungal burden phenotype. (A)** Maps of the disruption cassettes used to introduce upstream and downstream transcripts in the deletion mutant. pAP3 and pAP4 were constructed from pAC245 by replacement of either *orf19.2646* 5′-UTR or 3′-UTR, respectively. **(B)** mRNA level of Tn7 putative upstream and downstream transcripts. Results are expressed relative to the quantity measured for the isogenic wild-type strain. Each bar-plot corresponds to the mean of two biological samples measured in triplicates. **(C)** *G. mellonella* fungal burden for each individual larva. The fungal burden is expressed as a percentage relative to the mean of the wild-type group in the same experiment. All experiments were pooled in this graph. The mean value of each group is indicated with a black bar. The dotted line indicates the level of the mean of the wild-type strain set at 100%. Each mutant was tested at least twice using 10 larvae each time, except for the S-revertant which was tested only twice with 5 larvae (missing dots correspond to dead larvae). Statistical analyses were performed using a ROUT analysis to remove outliers (Motulsky and Brown, 2006), followed by a Mann–Whitney test to assess CFU differences relative to the wild-type strain (GraphPad Prism 7.02). The stars indicate the level of statistical significance: ^*^p < 0.05, ^***^p < 0.001.

After the identification of unexpected transcripts, their role in the fungal burden phenotype of a *C. albicans* strain was addressed. For this purpose, two plasmids (pAP3 and pAP4) were designed from the *orf19.2646* deletion cassette pAC245 (Figure 6A, for details see Material and Methods). The cassettes were inserted at the *orf19.2646* locus by double cross-over in a deletion mutant (ACY315). As the 3′-UTR of the downstream transcript was no longer present in the deletion mutant, this cassette was transformed into the *orf19.2646* heterozygous mutant (ACY297) to replace the remaining wild-type allele. Two strains: one carrying only the upstream transcript allele (ACY402; up-revertant), and another with the downstream transcript allele (ACY404; do-revertant) were thus obtained. A mutant with two transcripts was also constructed (ACY405; up+do-revertant). One allele would possess the upstream transcript allele, while the other would possess the downstream transcript allele. As a control, both constructs were transformed into SC5314 (data not shown). All strains were verified by southern-blot analysis (data not shown).

To verify that the new mutant strains ACY402, ACY404, and ACY405 expressed the upstream and downstream transcripts, we performed the same RT-qPCR as above (Figure 6B). The strain SC5314, the wild-type collection strain (WT collection), the S-revertant and the Tn7 mutants were used as controls. As expected, those strains carrying at least one wild-type allele of *orf19.2646* (SC5314 and WT collection), as well as the Tn7 mutant, displayed amplification of both the upstream and downstream regions. As previously observed, the S-revertant displayed amplification only for the upstream region. For the up-revertant, we observed a signal for the upstream probe, but it was 20 times reduced as compared to SC5314. For the do-revertant, we observed a signal for the downstream probe, but 8 times lower compared to SC5314 (Figure 6B). The strain carrying both the upstream and the downstream transcript alleles (up+do-revertant) showed signals for both the upstream and the downstream-probe which were 8- and 30-fold lower than SC5314, respectively. These results are in contrast to the level of expression measured in the Tn7 mutant, for which both signals were at least 10-fold higher than wild-type. All together, these data indicated that the reconstructed upstream and downstream transcripts were successfully produced, although to lower amounts as compared to wild-type (Figure 6B).

It was next investigated whether these transcripts were responsible for the low fungal burden observed in the *orf19.2646* Tn7 mutant, both in mice and in the mini-host model *G. Mellonella* (Amorim-Vaz et al., 2015). For this purpose, the ability of the different up- and do-revertants to invade larvae of *G. Mellonella* (Figure 6C) was tested. Regarding the Tn7 mutant, we observed a significantly reduced fungal burden as compared to wild-type as previously published (Amorim-Vaz et al., 2015). For both the up- and do-revertants, no significant change in fungal burden was observed as compared to wild-type strain. Nevertheless, when both the upstream and downstream transcripts were expressed together in the same strain (up+do-revertant), a significant reduction of fungal burden was measured (ANOVA adjusted *p* = 0.037; Mann-Whitney *p* = 0.0008). Fungal burden observed with the strain expressing both transcripts was 60% reduced when compared to wild-type, while a 90% reduction in fungal burden was observed with the Tn7 mutant. These data suggest that the presence of both transcripts is required to recapitulate a low fungal burden phenotype, although not at the same magnitude as for the Tn7 mutant.

### Deletion of the Tn7 upstream and downstream flanking region in the *orf19.2646* Tn7 mutant

**Figure 7:**
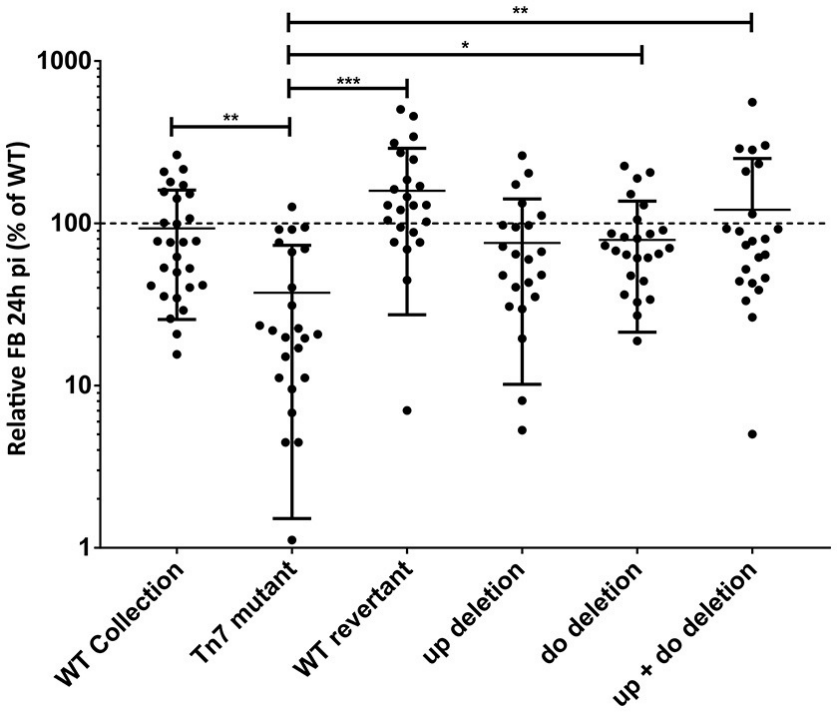
**Deletion of the Tn7 upstream and downstream regions and consequence on larvae fungal burden (FB)**. For each individual larva, the FB is expressed as a percentage relative to the mean of the wild-type group in the same experiment. All experiments were pooled in this graph. The mean value of each group is indicated with a black bar. The dotted line indicates the level of the mean of the wild-type strain set at 100%. Each mutant was tested at least twice using 10 larvae each time and once using 5 larvae (missing dots correspond to dead larvae). Statistical analyses were performed using a ROUT analysis to remove outliers (Motulsky and Brown, 2006), followed by a Kruskal-wallis (non-parametric ANOVA) analysis with a Dunn post-test to assess CFU differences relative to the wild-type strain (GraphPad Prism 7.02). The stars indicate the level of statistical significance: ^*^p < 0.05, ^**^p < 0.01, ^***^p < 0.001.

Unexpected transcripts of *orf19.2646* Tn7 mutant were deleted to determine the level of fungal burden phenotype in *G. mellonella*. Strains derived from the Tn7 mutant lacking either the Tn7 upstream, downstream or, both regions (ACY430, ACY422, and ACY428 respectively) were obtained. The genetic composition of the strains was verified by southern-blot (data not shown). *G. mellonella* were infected with the mutants and compared with larvae infected with the *orf19.2646* Tn7 mutant and the isogenic wild-type strain (Figure 7). Low fungal burden of the Tn7 mutant as compared to wild-type (adjusted *p* < 0.0001) was observed again in this model. The strains deleted for the Tn7 upstream or downstream regions also displayed a fungal burden higher than the Tn7 mutant (ANOVA adjusted *p* = 0.1, and *p* = 0.04, respectively), even if only the strain deleted for the downstream region gave a significant result. The strain deleted for both the upstream and the downstream regions (ACY428) displayed a fungal burden similar to the isogenic wild-type which was significantly higher than for the Tn7 mutant (Figure 7; ANOVA adjusted *p* = 0.0047). Taken together, these results confirmed the role of these two unexpected transcripts upstream and downstream the Tn7-UAU cassette in the low fungal burden phenotype observed in mice and *G. mellonella*.

## Discussion

The aim of this study was to analyse the Tn7 interruption mutation of the *orf19.2646 C. albicans* mutant in order to determine the cause of its low fungal burden phenotype observed *in vivo* which was not recapitulated by a null mutant. One possible explanation for this phenotype was that a truncated protein was formed as a result of the Tn7-UAU cassette insertion. Indeed, as previously described for several Tn7 mutants, we could demonstrate that a Tn7 upstream transcript was formed corresponding to a truncated form of the *orf19.2646* transcript. Surprisingly, we also found that downstream of the Tn7 cassette, another transcript was formed corresponding to a complete unexpected transcript. We determined the sequence of these two transcripts by RACE analysis. After reintroducing the transcripts into an *orf19.2646* deletion mutant, we observed that both the upstream and downstream transcripts were responsible for the low fungal burden phenotype observed with the Tn7 mutant. Similar results were obtained when the corresponding sequences for the 2 transcripts in the Tn7 *orf19.2646* mutant were deleted simultaneously. An exact determination of the insertion site of the Tn7-UAU cassette in this mutant was achieved which does not correspond to previous annotations by the TIGR consortium.

As previously described for other Tn7 mutants, the STOP codon of the unexpected upstream mRNA was found to be located within the Tn7 transposon. Indeed, Tn7 sequences (L or R) have been previously shown to contain stop codons in all the 3 frames (Lo et al., 2003). We were able to identify a putative PolyA signal (AUAAA) in the 3′-UTR of the transcript 103 bp upstream of the polyA sequence. It has been previously described that in contrast to the mammalian PolyA signal located -35 to -10nt upstream of the polyA sequence, the PolyA signal in yeast is more flexible in term of position and sequence (Moqtaderi et al., 2013). We were able to locate a putative TATA box with a non-canonical sequence 85 nucleotides upstream of the transcription site of this upstream transcript. However, initiation of transcription in yeast does not necessarily require a TATA box and no clear consensus sequence exists for an effective yeast TATA box (Basehoar et al., 2004).

Sequencing of the downstream transcript revealed that it was composed of part of the *orf19.2646* sequence. To our knowledge, the occurrence of unexpected transcripts downstream of a Tn7 sequence has not been previously described. We hypothesized that the Tn7 sequence was part of the promoter region. This was based on the observation that the coding sequence of the downstream transcript has a different reading frame than that of the wild-type gene, and that no gene locus has been documented in this region by the Candida Genome Database (CGD). The mRNA produced does not correspond to any known protein or protein domain. To verify the hypothesis that the Tn7 sequence was part of the promoter region, we could delete part of the transposon and test whether a transcript is still produced. In the meantime we could identify a putative PolyA signal (AUAAA) in this transcript situated 6 bases upstream of the PolyA sequence. As for the upstream transcript, we were unable to localize a canonical TATA box upstream of the transcription start site.

As previously described for other strains (Amorim-Vaz et al., 2015), the S-revertant strain did not appear to behave significantly differently from its isogenic wild-type strain after infection of *G. mellonella*, which is in contrast to the mouse experiments (Figures 3, 6C). Such results were attributed to the higher variability observed in *G. mellonella* than in mice, which might be due to the inbred lineages of mice in contrast to *Galleria* larvae. However, this result is in agreement with the up- and do-revertant results in *G. mellonella*. As for the S-revertant, the up-revertant was not sufficient to confer a significantly lower fungal burden in the *G. mellonella* larvae than for the wild type strain. The do-transcript effect is needed to obtain a phenotype sufficiently pronounced to be significantly different from the wild type strain in *Galleria*. However, the decrease of fungal burden observed with the Tn7 mutant was more pronounced than for the up+do-revertant (Figure 6C).

These results could be explained by the difference in expression of the up- and do-transcripts in both strains when compared to their respective wild-type strains (Figure 6B). The two strains do not have the same genetic background, one deriving from the BWP17 and the other from the SC5314 strain. Even though both parental strains are genetically related, as BWP17 is originally derived from SC5314, they may nevertheless have become genetically different by transformation over time. Thus these differences in parent strains could explain the observed discrepancies between the phenotypes of the Tn7 mutant and of the up+do revertant. No difference in sequence was detected in the promoter regions of *orf19.2646* in the BWP17 and SC5314 strains or in the Tn7 interruption mutant (data not shown). Therefore, the differences in the expression levels do not depend on polymorphism in the promoter region, but rather on another difference between these two strains.

Another explanation for the difference in fungal burden phenotypes might come from the *URA3* marker positioning. The auxotrophic marker *URA3* is known to play a role in the fungus colonization of its host (Brand et al., 2004). The two transcripts possess a wild-type copy of *URA3* that is located at its original locus, while in the Tn7 interruption mutant, *URA3* is located within *orf19.2646* genomic locus. This factor could therefore play a role in the *G. mellonella* fungal burden level in addition to the differences of mRNA expression.

In this study we describe how the use of the Tn7 transposon for mutagenesis could lead to artifacts in *C. albicans* as has been described previously for filamentous fungi (Lo et al., 2003), *S. cerevisiae* (de Jesus Ferreira et al., 2001), and *C. glabrata* (Borah et al., 2011). Indeed, we show that the Tn7 transposon promotes a premature polyadenylation of the interrupted gene. Surprisingly and for the first time, we also identified the presence a Tn7 downstream transcript. In this last case, the Tn7 sequence might play the role of a transcriptional activator. In consequence, the phenotypic effects observed in the screening are not due to the absence of the gene of interest but are rather due to the existence of chimeric transcripts.

One of the options to avoid such an artifact would be to screen at least 2 or 3 Tn7 mutants of the same gene and consider it a “stable” phenotype if all mutants display the same phenotype. This would reduce the number of false positives for a given phenotype. However, these insertion mutants may display different phenotypes to the deletion mutants due to the presence of truncated protein expression, and might reveal unsuspected cellular process implicated in the phenotype studied. In addition, transposon insertions might occur in an essential gene as described in a *C. glabrata* study (Borah et al., 2011) and may reveal functions of truncated versions of the gene. Finally the Tn7 might be inserted in predicted non-coding region but may display a phenotype, thus revealing non-annotated loci (de Jesus Ferreira et al., 2001).

In the current study, the two unexpected transcripts interfere with a biological pathway involved in virulence or at least in adaptation to the host. In the future, tagging these transcripts will allow us to confirm their translation into proteins and to visualize them within the cell. Further transcriptional analysis would allow us to understand which pathways are modulated by expression of those new proteins. All together this study identified two unexpected transcripts involved in reduced fungal burden. This opened new avenue of investigation on a yet unidentified virulence mechanism involved in the host invasion process.

## Author contributions

All authors were involved in the design of the studies and in the interpretation of the data. AP and FI carried out the majority of the experimental work, especially molecular biology experiments under the supervision of AC. AP and AC carried out the *Galleria* experiments. In addition to its involvement in the design of the studies, AC provided background information, strains and a critical input into the preparation of the manuscript. AP and AC wrote the manuscript.

## Funding

This project was supported by the Swiss National Science Foundation through grant number PMPDP3_13960 and by the Novartis Science foundation.

### Conflict of interest statement

The authors declare that the research was conducted in the absence of any commercial or financial relationships that could be construed as a potential conflict of interest.
