# Supplementary material for: Unexpected Transcripts in Tn7 orf19.2646 C. albicans Mutant Lead to Low Fungal Burden Phenotype In vivo
Source: Front Microbiol. 2017 May 16;8:873. doi: 10.3389/fmicb.2017.00873 (PMC5432668; doi:10.3389/fmicb.2017.00873)
Supplement: Supplementary file 1 [file DataSheet1.DOCX]

# File S1

## 1. Upstream transcript sequence

**
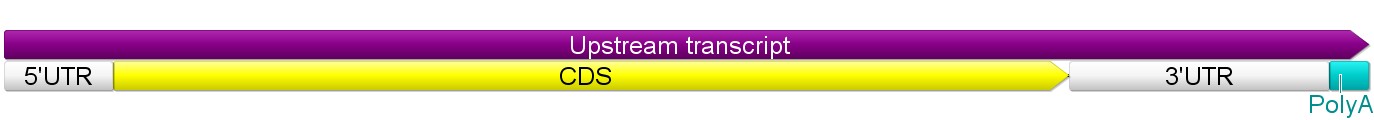
**

AACUUCCUCUUCAUAUUCUUUCAUUUAAUUAAUUAAUUAAUUGUAUCCCCCUCAACUAGCAGUUAGUUUUUUUUUUUUGUUUCAAAUAUACUUACUUUCAAUCAACGGAGAAAGUAAUCGAUUAUGGAUAAGACAAAUAGUCCAGGCAAAAAAGACAAUCGGUCUAGUGCAUCUCCUCAAAUACCGAAAAAACGGUCUAGAGUCCGACAACCAUUGAGUUGUUCAGUUUGCAGGAAAAGAAAACUGAAAUGUGAUCGGGCUCGUCCUUGUGGAACUUGUAUUAAAAAGAGUAUUGUACAUUUAUGUCAUUAUGAAGAUGAUAAUCGACCUCCCAUCAACCAUUUUCUACCCCCUGAACAACAAUUACACCCAACCCACAUUGACAAUAACGGAUAUAUAAUAACCGACCAACCACCGCCAAUACAUUAUCAAGACCCAUACAACAACCACAACCACAACCACCAUUUCCAGCAGCAGCAGCAGCAGCACCACCACAACACUAAUAAUGAUAGCAAUUUUGACCCAUCACUUCUGCAUAUACAAGCAAAUGGACAUAAUCAGUUUCAGCACCAACCAUUAUCAAGUCAUUCACCUCAGGGUAACCAUCAAUAUUUGCCAAUACCGCCACCACCACCGCCACCUCCACAACUGCAACAAUCACAACAUCCUCCUUCUAUUUCUCCAGCGGGGUAUAACUCUCUGAUAAAGAUGUCGCAGGCACAAAUCUCCAUUCCAACGCCACCACCGGCCACAAAAUCAUCUACUUCAACAUCAAAUCACAGUAGUCCAAACAGACCCCCUACUUCAUCAGGGCAAUCUAGUUACCACACCACCACUACCAACAAUUACUCUAAUUUCAAUCCUAGCACAGUUUCUAAUAUAAAUUCUAAACCAAAAUUGAAUUCAAUAAGUUUACCCUUACCACCACCUCCACCACCACCUUCUGUAACGACACCAUCUUUACCUAUGCCAUCCACCACAAAAUCAUCAAUAUCUGGCAUGAGUCUACAUCACGACAACACUUUUGGACAAGUUUCUAUUCCAUCUCCAAUCCCACCAAUAAAUGAAUUGUCACCAUCUUUACUGAGACUAAAAUCAUUAGGUUCAAACUCAGAUGGAGUGUUGUCACCAACUACAAUUGGAGUAAACGAUCUUCUUAAUCCAUCUAGAUCUAAUGGAGGCUAUAAUUGUGGGCGGACAAAAUAGUUGGGAACUGGGAGGGGUGGAAAUGGAGUUUUUAAGGAUUAUUUAGGGAAGAGUGACAAAAUAGAUGGGAACUGGGUGUAGCGUCGUAAGCUAAUACGAAAAUUAAAAAUGACAAAAUAGUUUGGAACUAGAUUUCACUUAUCUGGUUGGUCGACACUAGUGAUAAAAAAGGCCUGAUUUGGAUGGUAUAAACGGAAACAAAAAAAAGAGCUGGUACUACUUUCUUUAAAAUUAUUUUAUUAUUUGAUUUUAUUUAAUAGUAUAUAUUAUAUUUUGAACGUAGAUUAUUUUGUUGAAAAAAAAAAAAAAAAAAAAAAAAAAAAAAAAAAAAAAAAAAAA

In red are indicated the putative polyA signal

2. Downstream transcript sequence


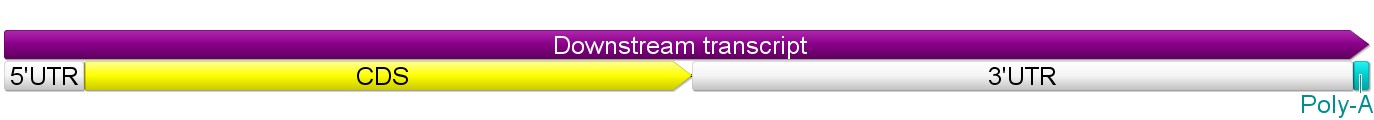


CAGGCGAUGAAAAUUUAUCACCAGUGGCAACAAAGAGACCAAAACUAGACAAUAAUGGUAAUGGAAACGCCAAUGGCAACGGUAACGGCAACAGCAGUGCAUCUUUAAAUACUGUAUCAAUGCCGAUGAACCGAUCAAAUUCUAAUGAUUCCAACUACUUAUCAAAUAAUGCCAAUUUCACUGAUGCAUUUGACAUGAUAUCGGAAAAUGAUAAACCAAGCAACAAUACCCCAUUGGAAACGUUUUCACCUCUGGUACAACAACAAUAUGAAAGUCCCAAGAAAACACCAGGAGAUAUGGCAUUUAUGCAAUCGUUUUAUAAUGGUGGACAAAAAAGACAGGAAUAUUAUAGAUUUGUUGAAGAUGAAGUUUCGAAAAUUUUGCCUGAUAAAACCAAUCUGUUUCAAUUAUUUUGCCGAUACUUUAGGUUUGUUAAUCCAUUUAUACAAAUAGUUGAUGAACAUGCAUUGUUAUUUGAUAUUAAUCCUAUACUCCCGAAAUUUUUGAAAUUUAAUCAUGAAAAAUUUACUGAAGUCAAGAUAAAAAGUGAAAAUGAUUUAAGAACAUUGGGGAUAUUUUUAUUGGUACUCAAACUUGGAUAUCAAACUAUGAUUCAUAACGAUAAUGAACAUAAUAAUUAUAAUGAACAAGAAUUGAGUAUUAUAGAUAGUAUGCAACAAUUGGAUAACCCCACUUUUAACCGAAUUAUCAAUUUGUGUAUUGCUGAUGGAUUAAUCACGGCCAGGUCAUCGUUCAAACUAGUUCAAUUAUUGGCAUUGUUAUAUCAUUAUAAAGGAAUGAGUCCUGAUGAUUCUCAUGGAUUAUCGAGUGCUGAUUCACAAAUUUUGUUGGGGACAAUAAUUAGACAUGCAUUUUCCAUUGGAUUAAACCGAGAUCCCACUCGUUACACCACAUUUGACAAUUUAGCGAAAAAUCAAGUAUUAAUUAAAACUUGGAGACAUCUUUGGUGGUUUUUAGUAGCCACCGAUGCCAUGAGUGCAUUGAAUAUUGAAUACUGGUUGUAAUUUGAAUGUAUCGAGUCUUGAUGGGUGUGAUGUUGAAUACCCGCACGUUUCAGAAGAUCCCACGGGUGAAAUGAACAAGAUAUAUGAAGUUUUAGCGAAAAUAUGUGAACAUUAUCGAAAUAUUGUCAAUAAGAUCAAUAAUUUACGACAAAAACCAAAAGUUGUGGAGAUUUUAAAAGAAACUAAUCAAAUGGAAAGAAUUUUUUUCGAUUUUUUCGGUAAAGAUUUUUUCAAAGAUGUGGUUUGUAAACCUGCUAAAGAACCGACUAAUGGGAAUGGAUUUGAAGAAGCUAGUAAAGAACAUAUGGAAAAAGUUGUUAAAGUUUUCAAAUAUUGUUUGUUUAUUCAAUUGAGAACCAAUUUAUCGGGGAUGUAUUAUAAAAUUGCCAUACAUUAUGAAAAUGAAUAUGAUAAAUCCAAGACCCCUUCUAUGAAAGCUGGGAUUGAAUUAUUUAAAAUUUAUAUUAAAAGUGUUGUACAAUUGGUUUAUAUCAUGUCUUAUGUUUUGGAUAAUUCUGUAUAUUUAUUUGGGAAAAAUUUUGAUUAUAUGUUAACGGCAUCGAAUGAAAGAUAUAUGAUUAAGACUCAUUCAUUUUUAACAUCAUUUUUCGUUAGAUUAUUACAUCAGAAAAAGGGACUGUCAUUUAAAGUUUUCAAAGAAAUCAGUUAUAUGUCACGAUUAGAAUGUAUUAAUAAUUUAUUUGAUAUUGUAUUGGAAGAUGUUGAAUUAUUUGUUGGUGAUUUCCGAAGAUUAUCUAAGACAUAUAUCAAUUCUUAUCGAUUAUAUAUUAUUACUUUUAUUGUUUUACGACAAUCAAUUGAUAAUUCUGACGCAUUUUUCGAAAAAGCCGCUAGUGAUCAACUGUUUUUCCAUCAAGGGACCAAUAUGAUUGAAUUUUUUUCACAACAAGAAUUAAAUCAUUUAUGCCGAUUAUGUAGAGAUUGGAGAAAUAUUAAAGAAGCUCAAAAAAAAUAUAAAGAUGCUAAAAAAAAAAAAAAAAAAAAAAA

In red are indicated the putative polyA signal
